# GWAS quality score for evaluating associated regions in GWAS analyses

**DOI:** 10.1093/bioinformatics/btad004

**Published:** 2023-01-18

**Authors:** Swapnil Awasthi, Chia-Yen Chen, Max Lam, Hailiang Huang, Stephan Ripke, C Anthony Altar

**Affiliations:** Department of Psychiatry and Psychotherapy, Charité - Universitätsmedizin, Berlin 10117, Germany; Biogen, Cambridge, MA 02142, USA; Stanley Center for Psychiatric Research, Broad Institute of MIT and Harvard, Cambridge, MA 02142, USA; Stanley Center for Psychiatric Research, Broad Institute of MIT and Harvard, Cambridge, MA 02142, USA; Analytic and Translational Genetics Unit, Massachusetts General Hospital, Boston, MA 02114, USA; Department of Psychiatry and Psychotherapy, Charité - Universitätsmedizin, Berlin 10117, Germany; Stanley Center for Psychiatric Research, Broad Institute of MIT and Harvard, Cambridge, MA 02142, USA; Analytic and Translational Genetics Unit, Massachusetts General Hospital, Boston, MA 02114, USA; Splice Therapeutics, Germantown, MD 20876, USA

## Abstract

**Motivation:**

The number of significantly associated regions reported in genome-wide association studies (GWAS) for polygenic traits typically increases with sample size. A traditional tool for quality control and identification of significant regions has been a visual inspection of how significant and correlated genetic variants cluster within a region. However, while inspecting hundreds of regions, this subjective method can misattribute significance to some loci or neglect others that are significant.

**Results:**

The GWAS quality score (GQS) identifies suspicious regions and prevents erroneous interpretations with an objective, quantitative and automated method. The GQS assesses all measured single nucleotide polymorphisms (SNPs) that are linked by inheritance to each other [linkage disequilibrium (LD)] and compares the significance of trait association of each SNP to its LD value for the reported index SNP. A GQS value of 1.0 ascribes a high level of confidence to the entire region and its underlying gene(s), while GQS values <1.0 indicate the need to closely inspect the outliers. We applied the GQS to published and non-published genome-wide summary statistics and report suspicious regions requiring secondary inspection while supporting the majority of reported regions from large-scale published meta-analyses.

**Availability and implementation:**

The GQS code/scripts can be cloned from GitHub (https://github.com/Xswapnil/GQS/). The analyst can use whole-genome summary statistics to estimate GQS for each defined region. We also provide an online tool (http://35.227.18.38/) that gives access to the GQS. The quantitative measure of quality attributes by GQS and its visualization is an objective method that enhances the confidence of each genomic hit.

**Supplementary information:**

Supplementary data are available at *Bioinformatics* online.

## 1 Introduction

**Fig. 1.**
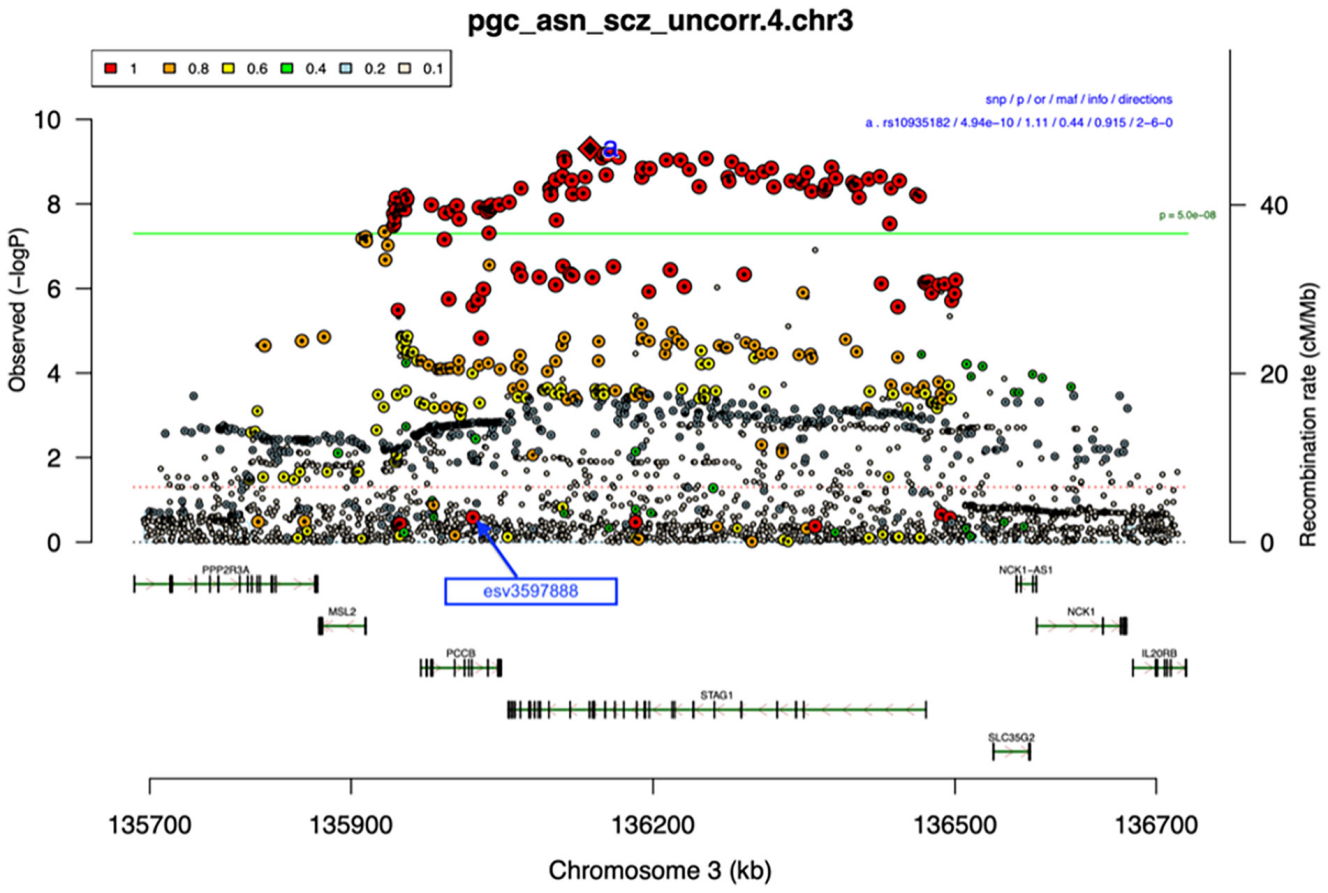
Traditional representation of genomic regions associated with a trait. This example is from an uncorrected (unpublished) PGC Asian schizophrenia GWAS^7^. The x axis represents the chromosomal position (in kb), and the y axis represents the degree of association measured as −log_10_(*P*). The green continuous line is the genome-wide significance level *P* = 5 × 10^−8^. Each dot is a SNP, and the dot size are proportional to LD between each SNP and the index SNP of the region, represented by a red diamond and labeled ‘a’. Coloring is based on the extent of LD to the index SNP measured as *R*^2^. The color coding for *R*^2^ is given in the legend in the upper left of the figure. In this region plot, there are several SNPs in high LD with the index SNP (colored in red), which are unexpectedly showing no significance (*P* > 0.05) ‘esv3597888’, which will be shown in later plots, is marked with an arrow (A color version of this figure appears in the online version of this article)

In the first decade of their widespread use, genome-wide association studies (GWAS) have identified well over 300 000 single nucleotide polymorphisms (SNPs) associated with various traits (https://www.ebi.ac.uk/gwas/docs/file-downloads). For example, the GIANT consortium identified 3290 independent SNPs associated with the height of 700 000 individuals via a GWAS meta-analysis (Yengo *et al.*, 2018). The growing use of GWAS meta-analyses for polygenic traits like height presents a daunting problem to interpret or verify the accuracy of the rapidly increasing number of reported SNPs. At present, visual inspection and subjective impressions of SNP *P*-values are used to interpret whether there is an expected clustering of SNPs around an associated index SNP in a genomic locus (e.g. Fig. 1). These subjective, manual estimates depend on the experience of the analyst and potentially create an incomplete or erroneous report of associated genes. There is a need for an objective, quantitative and reliable method to measure the extent to which phenotype is associated with variations throughout each genomic region and to identify spurious signals in an automated way.

Here, we describe a novel method, the GWAS quality score (GQS), that exploits the relationship between test statistics and linkage disequilibria within an associated genomic locus in GWAS (Bulik-Sullivan *et al.*, 2015; Pritchard and Przeworski, 2001; Sham *et al.*, 2000; Yang *et al.*, 2011). Our method systematically classifies and identifies the SNPs that do or do not follow this relationship for each associated region in GWAS. It assigns a quality score to such genomic regions and flags them for secondary inspection. This objective, quantitative evaluation of the significance of SNP clusters simplifies the interpretation of results by ascribing confidence levels to the SNP cluster and the locus in which the cluster resides. Moreover, the GQS reinforces confidence in true positive associations and to the whole-genome GWAS summary statistics.

The detection of outliers by the GQS method is mostly driven by technical artifacts at the level of SNP calling—namely, failing cluster algorithms for allele intensities and inconsistent merging of data from distinct sources. While we show here that the GQS method can evaluate single-cohort analyses, it is especially powerful in evaluating popular meta-analyses, where due to incomplete SNP overlap, different sample sizes in correlated SNPs are common. Similar single-cohort outlier detection with traditional approaches is feasible only with raw genotype access, while GQS summary statistic outlier detection is similar but not identical to DENTIST (Chen *et al.*, 2021). In summary, the GQS method can detect outliers that were undetected by existing QC metrics (including DENTIST), while the evaluation of meta-analysis summary statistics has not been described by published methods.

As shown by the present results, the single GQS value identifies false positive and false negative results and confirms true positive and true negative results. We first trained and tested our method on false positive hits from simulated genotype data and unpublished meta-analysis data with pre-identified spurious associations. We then analyzed 244 genomic regions from unpublished schizophrenia GWAS, and finally on many verified associations from published results of the Psychiatry Genomics Consortium (PGC) (Sullivan *et al.*, 2018), GIANT consortium (Yengo *et al.*, 2018), Social Science Genetic Association Consortium (SSGAC) (Okbay *et al.*, 2016) and coronavirus disease 2019 (COVID-19) host genetics initiative (COVID-19 Host Genetics Initiative *et al.*, 2021).

## 2 Materials and methods

### 2.1 Overview of the method

The principle guiding the GQS method is that non-associated SNPs that are in high linkage disequilibrium (LD) with a significantly associated ‘index’ SNP are suspicious. The GQS method calculates a quality score and flags suspicious loci for secondary inspection.

**Fig 2.**
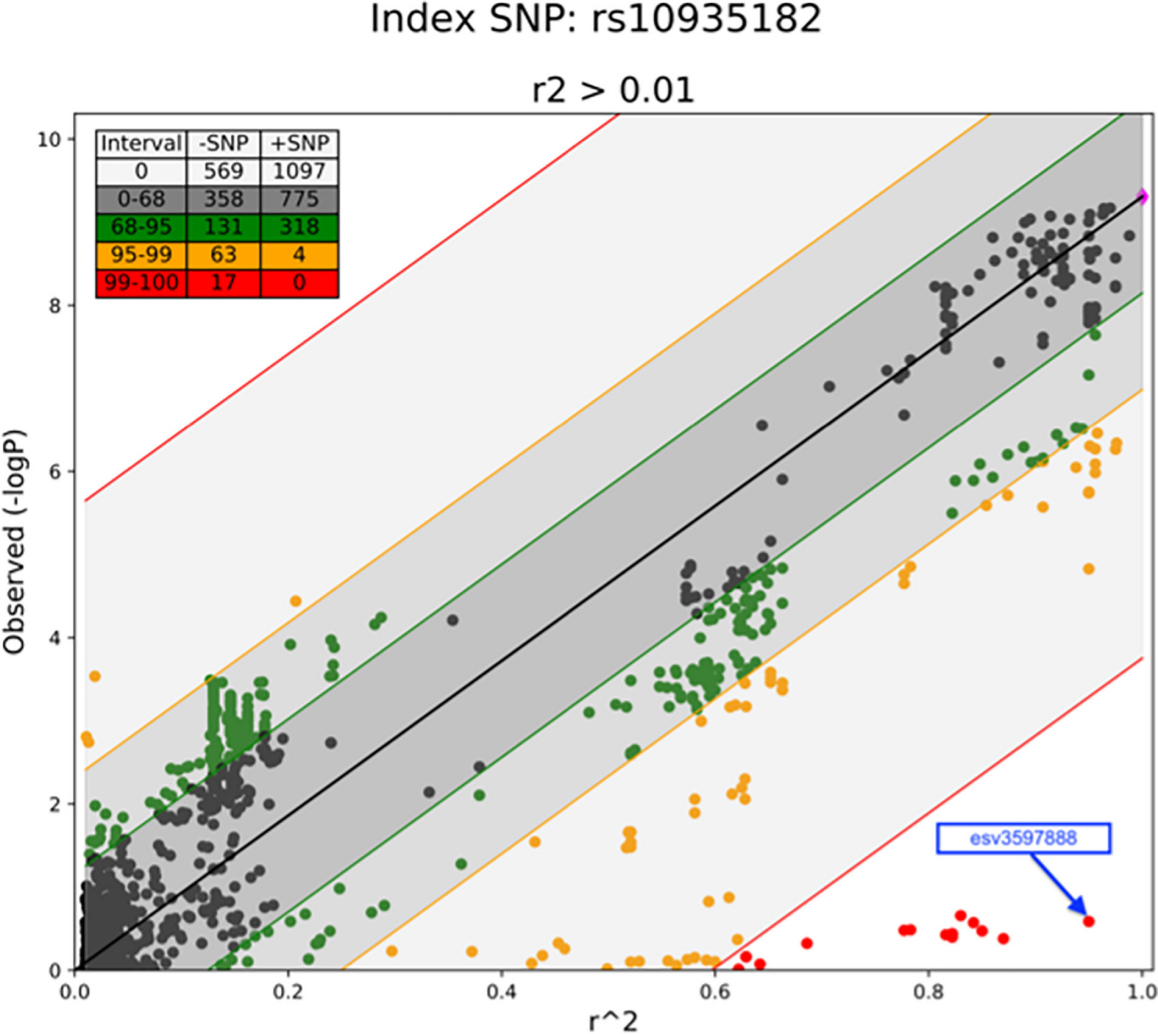
GQS scatter plot for the data shown in Figure 1. The x axis is the extent of LD (*r*^2^) of each SNP (single dot) with respect to the index SNP (magenta dot, at *r*^2^ = 1) and the y axis is the significance of association to the trait, measured as −log_10_(*P*). The black regression line represents the well-known correlation between the statistical significance and LD (Bulik-Sullivan *et al.*, 2015; Pritchard and Przeworski, 2001; Sham *et al.*, 2000; Yang *et al.*, 2011). The extent that SNPs agree with the theoretical regression line is stratified according to the shaded regions. The darkest gray shaded region encloses 68% of SNPs (black dots). The medium grey shaded region contains 68–95% of SNPs (green dots) and the lightest shaded region engulfs 99% of SNPs (orange dots). In this example, 1% of SNPs (red dots) lie farthest from the regression. The table in the upper left corner shows the number of SNPs in each interval that falls below (−SNP) or above (+SNP) the regression line (A color version of this figure appears in the online version of this article)

The first step to calculate the GQS is to identify the gene locus and index SNP in a classic regional association plot (Fig. 1). The index SNP is the one with the lowest *P*-value within a genomic region. The GQS then estimates the LD between each SNPs in the region and the index SNP using the appropriate population reference genome. SNPs in linkage equilibrium (*r*^2^ of <1%) are excluded to reduce noise in the statistics. Then, the GQS method delineates a straight line from the index SNPs to the origin in a 2D space of the negative log of *P*-value and LD-r2 (e.g. see Fig. 2).

The regression line represents the well-known correlation between the test statistics and LD when the regional index SNP is associated with the trait and the underlying LD information is correct (Bulik-Sullivan *et al.*, 2015; Pritchard and Przeworski, 2001; Sham *et al.*, 2000; Yang *et al.*, 2011). The GQS method then categorizes the SNPs into those that lose the signal of statistical significance (positioned below the regression line) or gain signal (positioned above the regression line) beyond expectation. For visualization purposes, the methods also segregate SNPs into the areas within which 68%, 95% and 99% of data points (SNPs) lie from the regression line. It calculates the residuals of each SNP (i.e. data points) as the difference between the value predicted by the regressor and the empirically observed value (Equation (1)). Then, the ratio S between each residual (e in Equation (1)) and the predicted values for each is calculated (Equation (2)).

(1) $$e=\;p-\;\hat{p}$$

*p* = observed – log_10_ of *P*-values

$\hat{p}\;$ = expected – log_10_ of *P*-values

(2) $$S=\frac{e}{\hat{p}}.$$

This ratio *S* represents a proportion of signal gained (a positive ratio) or signal lost (a negative ratio) for each SNP above or below the regression. Thus, for the measure of association between a genomic region and a trait, *S* reflects the deviation of statistical significance of each SNP in that region beyond what would be predicted by its LD with the index SNP. In the calculation of GQS, we do not incorporate SNPs that gain signals since these likely reflect one or more additional, independent associations within the same genomic location. The existence of such additional association can be evaluated by a reanalysis of the data by choosing a different index SNP (Supplementary Fig. S8).

**Fig. 3.**
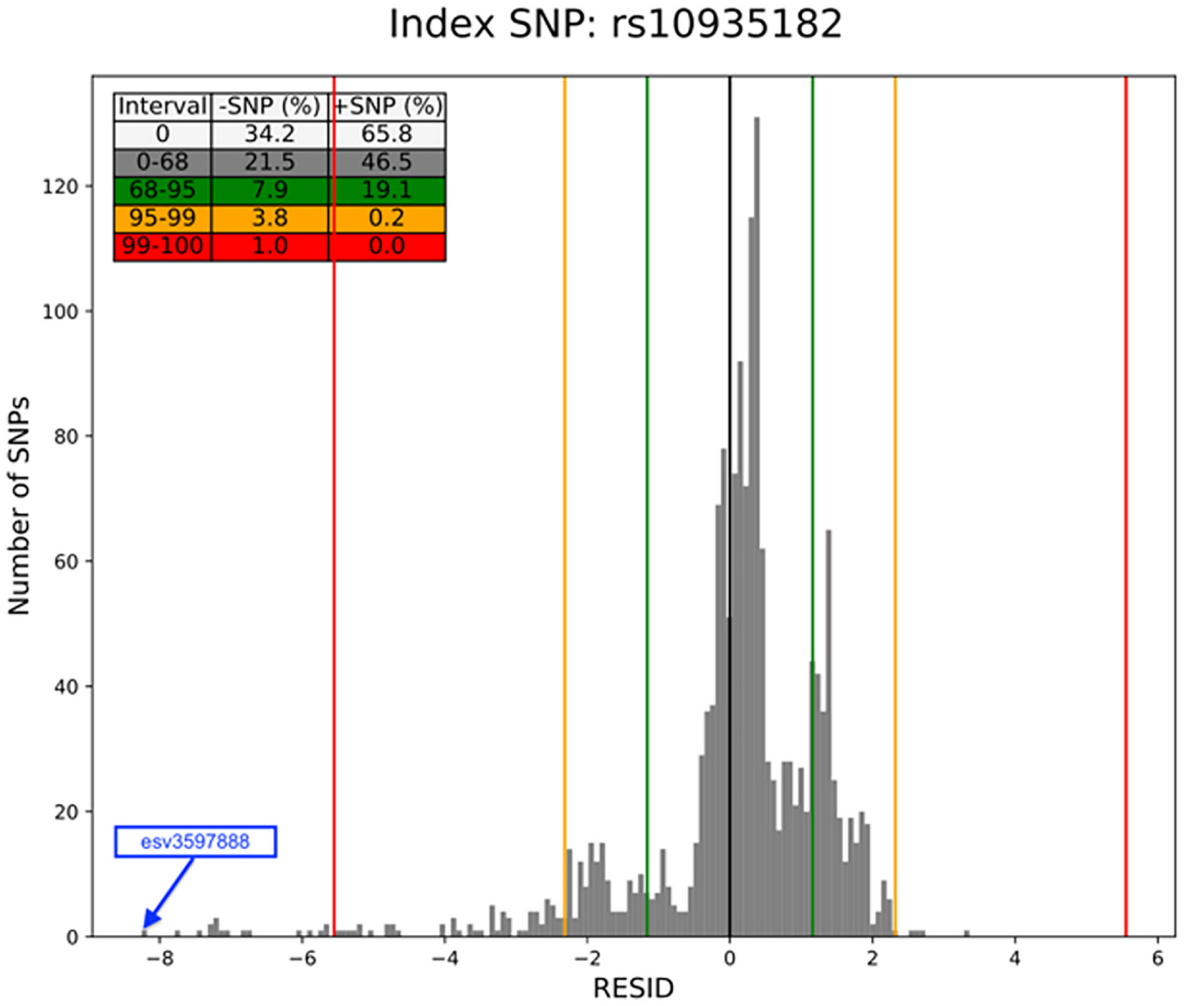
Histogram of the ratio of residuals obtained as per Equation (2). Values obtained from Equation (2) are plotted on the x axis and their number is given by the y axis. The thick black line at x = 0 segregates the SNPs into the ones losing (negative, left of 0) and gaining signals (positive, right of the 0). The green, orange and red lines further segregate the SNPs into those losing > 68, 95, and 99% signal, respectively. The table in the upper left corner shows the number of SNPs in the corresponding interval. For compatibility with Figure 2, we illustrate percentages instead of absolute numbers (A color version of this figure appears in the online version of this article)

**Fig. 4.**
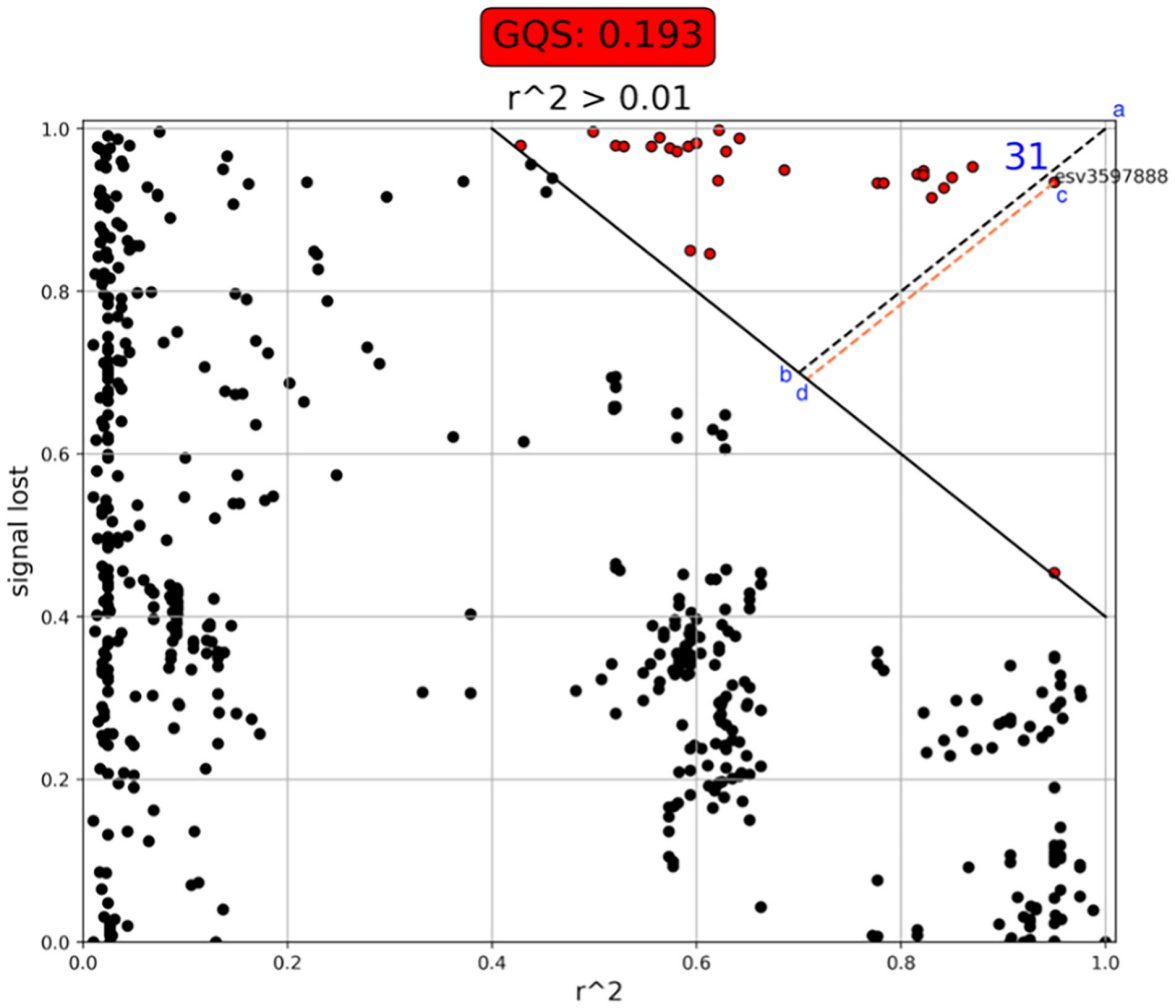
GQS plot. The x axis is the LD value with respect to the index SNP (*r*^2^) and the y axis is the signal loss calculated as described in the method and Equation (2). The solid black diagonal line is the linear drop threshold for the GQS, above which the significance reported for a SNP drops more than the expected significance drop based on its LD. The dotted black line (from blue points a to b) displays the worst-case scenario (i.e. a 100% loss of signal for a SNP that is in 100% LD with the index SNP). The distance from blue points c to d shows the biggest signal loss in the region (here ‘esv3597888’, which is an indel). These values are used to calculate the GQS based on Equation (3). (A color version of this figure appears in the online version of this article)

The distribution of all SNPs so tested reveals the extreme SNPs that show lower significance than the proportional loss expected from their LD with the index SNPs. The naive method is to simply identify SNPs that lose the most signal, such as more than 75% of signal than expected, and examine those for discrepancies (e.g. Fig. 3). In practice, however, we observed that the SNPs with lower LD-r2 deviate more in proportion to the prediction line than those in higher LD-r2. For this reason, such SNPs with lower LD-r2 and higher variability are not as crucial to assessing GWAS quality as are the SNPs with strong LD-r2 and lower significance (Fig. 4). Thus, the GQS method employs a linear function to classify each SNP with LD-r2 > 0.4 as an outlier or non-outlier for the GQS analysis.

The GQS drop threshold, shown as a solid black diagonal line in Figure 4, is a linear function that varies from a 100% loss in signal at 40% of LD-r2 to a 40% loss in signal at 100% LD-*r*2. We consider the excessive variance for LD-r2 values of <40% to provide insufficient signal to be considered informative for the GQS method. The quality of a genomic region depends critically on (i) the absence of suspicious SNPs above this linear threshold and (ii) how far the outliers SNPs are to the worst-case scenario. The GQS method estimates the ratio between the SNP with the maximal signal loss in the region (distance d–c in Fig. 4) and the worst-case scenario of losing 100% of the signal (distance a–b in Fig. 4). To summarize, the ratio assesses how close the strongest outlier SNP gets to the ‘worst-case scenario’ of total signal loss. This evaluates a quantitative GQS score for the region.

(3) GQS=1-Max ⁡signal loss in the region (distance from c to d)Worst case scenario (distance from a to b)

For the example in Figure 4, this translates to GQS = 1 − (0.342/0.424) = 0.193. The denominator has a constant value, $\sqrt{0.18},$ representing the worst-case scenario distance of a hypothetical SNP in complete linkage equilibrium (*r*^2^ = 1.0) to the index SNP while without any significance.

### 2.2 Overview of availability and web application

The GQS code/scripts can be cloned from GitHub (https://github.com/Xswapnil/GQS/). The analyst can use whole-genome summary statistics to estimate GQS for each defined region. It is also implemented in the Ricopili pipeline (Lam *et al.*, 2020) for GWAS. The web-based application for GQS is available on a Google cloud server (http://35.227.18.38/). The application has a user-friendly interface and does not require an understanding of UNIX.

### 2.3 Application on simulated data

The results from a GWAS of a hapgen (Su *et al.*, 2011) simulated cohort consisting of 464 cases and 516 controls with technical interventions were obtained and described before (Lam *et al.*, 2020), a broad description is also presented in Supplementary Section S1. We conducted the GQS method to analyze the association of over 1500 false-positive associations for these data.

### 2.4 Application of GQS on PGC SCZ wave3

The GQS method was tested on GWAS summary statistics for 244 genomic regions associated with schizophrenia (SCZ) obtained from the PGC Schizophrenia workgroup (The Schizophrenia Working Group of the Psychiatric Genomics Consortium et al., 2020).

### 2.5 Application on pre-cleaned PGC SCZ Asian analysis

Two sets of summary statistics were obtained from the PGC analysts who were responsible for the SCZ Asian analysis. One set, shown in the left half of Table 1, includes pre-publication summary statistics, for which unusual regional association patterns were identified by visual inspection of 20 significantly associated loci. These underlying data were not published as such, because visual inspection led to further cleaning of the data before publication. GQS for the cleaned final summary statistics is shown in the right half of Table 1 (Lam *et al.*, 2019).

**Table 1.** Comparison of GQS between all the significant regions from uncorrected/unpublished Asian SCZ GWAS and corrected/published Asian SCZ GWAS

	Uncorrected/unpublished GWAS				**Corrected/published GWAS**			
CHR	Index SNP	*P*-value	Outier	GQS	Index SNP	*P*-value	Outler	GQS
1	rs4660761	3.07E−09	5	0.612	rs4660761	2.97E−09	0	1
2	rs848293	1.50E−17	4	0.558	rs848293	1.69E−17	0	1
2	rs17592552	2.24E−13	6	0.618	rs17592552	2.11E−13	0	1
3	rs2073499	1.37E−13	5	0.627	rs2073499	1.40E−13	0	1
3	rs10935182	4.94E−10	31	0.193	rs10935182	4.78E−10	1	0.993
3	rs4856763	1.78E−10	6	0.71	rs4856763	1.80E−10	0	1
3	rs13096176	3.61E−09	17	0.037	rs13096176	3.45E−09	0	1
4	rs6832165	3.63E−08	1	0.673	rs6832165	3.58E−08	0	1
4	rs13142920	4.70E−09	2	0.482	rs13142920	4.80E−09	0	1
6	rs4479913	1.51E−10	8	0.197	rs4479913	1.49E−10	0	1
7	rs320696	3.46E−09	0	1	rs161336	3.56E−09	0	1
8	rs11986274	1.27E−08	17	0.185	rs11986274	1.28E−08	2	0.79
8	rs298182	1.35E−08	11	0.028	rs298198	1.38E−08	0	1
10	rs4147157	1.25E−15	8	0.498	rs4147157	1.34E−15	0	1
12	rs10861879	1.35E−08	1	0.64	rs10861879	1.27E−08	0	1
12	rs1984658	2.76E−14	29	0.4	rs1984658	2.97E−14	0	1
13	rs9567393	1.11E−09	3	0.165	rs9567393	1.17E−09	0	1
17	rs9890128	2.55E−09	3	0.597	rs9890128	2.72E−09	0	1
18	rs55642704	3.46E−11	2	0.92	rs2277725	3.36E−11	1	0.957

*Note*: GQS was carried out using default boundary of 100% loss in signal at 40% of LD-r^2^ to 40% loss in signal at 100% LD-r^2^.

### 2.6 Application on other published results

We downloaded publicly available summary statistics for various complex traits and applied the GQS method on the significant loci. We accessed summary statistics from a GWAS of adult height (*N* ∼ 700 000) (Yengo *et al.*, 2018), neuroticism (*N* = 170 911) (Okbay *et al.*, 2016) and educational attainment (*N* = 293 723) (Okbay *et al.*, 2016). We also tested on the three main GWAS summary statistics from the recently published COVID-19 host genetics initiative (release 5) (very severe respiratory confirmed COVID versus population, A2; hospitalized COVID versus not hospitalized COVID, B1 and COVID versus population, C2) (COVID-19 Host Genetics Initiative *et al.*, 2021).

### 2.7 Targeted region GQS from PGC SCZ

We obtained GRCh37/h19 location of various muscarinic and other annotated G protein-coupled receptors. Then we pulled out association data ±50 kb of each receptor’s location from PGC SCZ GWAS (The Schizophrenia Working Group of the Psychiatric Genomics Consortium *et al.*, 2020) and performed GQS for receptors most commonly targeted by antipsychotic agents (Table 2) and another 26 of the most highly GWAS-significant receptors (Table 3).

**Table 2.** GQS of muscarinic receptors and two prominent receptor targets for antipsychotic drugs, from PGC SCZ wav3 GWAS

Gene	CHR	Index SNP	*P*-values	Outliers	GQS
DRD2	11	rs2514218	6.46E−15	0	1
CHRM4	11	rs6485682	1.26E−15	0	1
CHRM2	7	rs56118357	2.36E−04	0	−1
CHRM1	11	rs78266386	2.71E−05	0	−1
CHRM3	1	rs112507592	7.90E−05	0	1
HTR2A	13	rs73175511	6.50E−04	0	−1
CHRM5	15	rs7170866	1.07E−03	0	1

*Note*: Note that reference SNPs were statistically significant only for DRD2 and CHRM4.

**Table 3.** GQS of G protein-coupled receptor regions from PGC SCZ wave3 GWAS

Gene	CHR	Index SNP	*P*-values	Outliers	GQS
GABBR1	6	rs3131856	2.08E−32	0	1
MAS1L	6	rs2206853	1.74E−31	0	1
GRM3	7	rs6943762	6.30E−17	0	1
GRM1	6	rs2206956	2.51E−09	0	1
CRHR1	17	rs35631660	3.92E−09	0	1
MCHR1	22	rs133047	1.73E−08	0	−1
OPRD1	1	rs533123	1.87E−08	0	1
LPAR2	19	rs7245672	2.32E−08	0	1
GABBR2	9	rs10985811	2.53E−08	0	1
GPR182	12	rs703848	4.18E−08	0	1
TAS1R1	1	rs14057	6.11E−08	0	1
GPR135	14	rs113239787	2.46E−07	0	1
MC1R	16	rs4785751	1.54E−06	0	1
GPR52	1	rs12565430	1.75E−06	0	1
GPR25	1	rs143917380	3.81E−06	0	1
HCAR1	12	rs80220833	3.86E−06	1	0.842
HCAR3	12	rs80220833	3.86E−06	1	0.842
HCAR2	12	rs10466883	4.49E−06	1	0.843
CELSR3	3	rs9878063	7.09E−06	0	1
HTR1A	5	rs749100	8.33E−06	0	1
LGR4	11	rs6484313	7.59E−05	0	1
GRM2	3	rs2518461	1.92E−04	0	1
GPR62	3	rs111632417	2.00E−04	0	−1
CMKLR1	12	rs4964667	2.29E−04	0	−1
NMUR2	5	rs116694426	2.70E−04	0	−1

*Note*: DRD2 and CHRM4 are not included here as they are shown in Table 2.

## 3 Results

### 3.1 GQS interpretation

Based on results obtained with simulated and robust real data, we recommend the following interpretation for GQS. In general, any region with one or more SNPs in the critical area above the GQS drop threshold and a GQS value <1 will require further investigation.

### 3.2 Simulated data

A total of 1819 independent regions with genome-wide significant index SNPs were artificially enriched for spurious results resulting from introduced technical artifacts. Among these, 1526 were detected with one or more problematic SNPs and were flagged by the GQS method as suspicious regions (Supplementary Fig. S1 and Table 4). Further investigation identified technical biases (e.g. high missingness rates per SNP) in all of these.

**Table 4.** Interpretation of GWAS significance based on GQS results

Number of outlier SNPs	GQS	Interpretation within the locus of interest
0	1	No extreme outliers. High statistical and empirical confidence throughout the LD region.
>1	<1	The regions with GQS <1 require a secondary inspection, the closer that GQS gets to 0, the more extreme the strongest outlier is, and other outliers are probably present.
0	−1	The negative value (−1) signifies that there are no SNPs to support the LD pattern with the index SNP (*r*^2^ > 0.4)

### 3.3 PGC SCZ results

GQS analysis revealed 244 loci from the SCZ GWAS that contained at least one SNP with genome-wide significance (*P* < 5 × 10^−8^). Among these associated regions, 239 produced a GQS of 1 (Supplementary Table S2), 20 had a GQS value of −1, and one genomic region had a GQS of 0.97. In summary, the whole-genome summary statistics for these regions did not reveal any suspicious loci (Supplementary Fig. S2).

### 3.4 PGC SCZ Asian analyses, pre-QC and post-QC

We applied the GQS method on 19 associated independent genomic regions that were identified in an uncorrected and unpublished Asian SCZ GWAS meta-analysis. The GQS method flagged 18 suspicious regions and correctly tagged the problematic SNPs.

In the cleaned, published GWAS, 17 of the 19 regions with genome-wide significance showed a perfect GQS score of 1. The three remaining regions with a GQS of <1 (0.93, 0.79 and 0.957) were inspected and found to be acceptable after scrutiny by Lam *et al.* (2019) (Supplementary Fig. S3a and b).

### 3.5 GQS on published GWAS

We applied GQS on publicly available genome-wide significant summary statistics for adult height, education attainment and neuroticism obtained from the following GWAS studies:

1. Height GWAS (Yengo *et al.*, 2018): Out of 1486 significant and independent regions associated with height GQS could flag 189 regions (182 with GQS of −1 and 7 with GQS < 1) that need to be scrutinized (Table 5 and Supplementary Fig. S4).
2. Education attainment GWAS (Okbay *et al.*, 2016): Two out of 73 significant hits showed a GQS value <1 (Table 6 and Supplementary Fig. S5) and five showed a GQS value of −1.
3. Neuroticism GWAS (Okbay *et al.*, 2016): Three out of 10 significant regions were flagged as suspicious. One with a GQS of 0.365 (Table 7 and Supplementary Fig. S6) and two with a GQS value of −1.
4. COVID GWAS (COVID-19 Host Genetics Initiative *et al.*, 2021): The publicly available data differed from the published manuscript regarding sample size, so the significant finding might vary with the publication. In total, we observed 16 GWAS loci for the three phenotypes (very severe respiratory confirmed COVID versus population A2; hospitalized COVID versus not hospitalized COVID, B1; and COVID versus population C2). Among these, 13 were flagged by our method, one with a GQS of −1 and for the other 12 the outlier SNPs ranged from 1 to 185 (Table 8 and Supplementary Fig. S7a–c).

**Table 5.** Seven regions out of 1487 from height GWAS that had GQS <1 at the default boundary of 100% loss in signal at 40% of LD-r^2^ to 40% loss in signal at 100% LD-r^2^

CHR	Index SNP	*P*-values	Outliers	GQS
6	rs9370463	3.90E−10	29	0.725
3	rs720390	5.80E−109	12	0.83
13	rs9530328	5.90E−10	1	0.885
4	rs7675744	5.80E−295	1	0.935
3	rs250405	3.00E−14	15	0.957
16	rs3112686	1.80E−11	1	0.957
22	rs4253755	9.70E−18	1	0.983

**Table 6.** Two regions out of 72 from education attainment GWAS that had GQS < at the default boundary of 100% loss in signal at 40% of LD-r^2^ to 40% loss in signal at 100% LD-r^2^

CHR	Index SNP	*P*-values	Outliers	GQS
16	rs8049439	2.69E−09	1	0.822
18	rs62100767	1.11E−08	1	0.852

**Table 7.** One suspicious region out of 11 in neuroticism GWAS that had GQS <1 at the default boundary of 100% loss in signal at 40% of LD-r^2^ to 40% loss in signal at 100% LD-r^2^

CHR	Index SNP	*P*-values	Outliers	GQS
17	rs193236081	6.26E−11	61	0.365

**Table 8.** Twelve regions out of 16 from COVID-19 GWAS on three distinct phenotypes (release 5) that had GQS <1 at the default boundary of 100% loss in signal at 40% of LD-r^2^ to 40% loss in signal at 100% LD-r^2^

CHR	Index SNP	*P*-values	Outlier	GQS	Phenotype
12	rs10860891	2.60E−08	33	0.01	A2
7	rs622568	1.42E−08	81	0.243	A2
6	rs111837807	4.71E−12	185	0.258	A2
19	rs11085727	2.51E−08	2	0.702	A2
3	rs35081325	2.70E−49	15	0.73	A2
21	rs12482060	3.66E−09	8	0.762	C2
12	rs10774671	2.09E−11	1	0813	C2
21	rs13050728	1.30E−14	10	0.832	A2
19	rs2109069	4.17E−21	1	0.855	A2
12	rs2269899	3.31E−13	5	0.863	A2
17	rs77534576	8.60E−09	3	0.885	A2
19	rs2109069	2.76E−08	1	0.972	C2

*Note*: The three phenotypes are very severe respiratory confirmed COVID versus population (A2), hospitalized COVID versus not hospitalized COVID (B1) and COVID versus population (C2).

### 3.6 Targeted region GQS from PGC SCZ

GQS on targeted regions from seven neurotransmitter receptors (Table 2) and another 22 G protein-coupled receptors (Table 3) were found to be 1 for 22 regions and −1 for seven regions. Only one genomic region spanning the three genes HCAR1, HCAR2 and HCR3 showed a GQS of 0.842.

## 4 Discussion

The statistical tool described here, the GQS, provides a quantitative measure of whether the SNPs in a genomic locus are consistently associated with the phenotype, as predicted by their LD structure. GQS values range from 0 to 1.0, where 0 has no consistent association with phenotype, to 1.0, with a highly consistent association. GQS can automatically identify problematic regions among hundreds of associations that might not be evaluated correctly or easily by the traditional subjective, visual inspection. Flags for such regions warrant a secondary inspection. Conversely, GQS values of 1.0 indicate high confidence in the validity of a reported region because the statistical significance of SNPs is in reliable concordance with the underlying LD pattern. As shown in Table 1, GQS can automatically identify suspicious association patterns within regions (e.g. due to technical artifacts).

One advantage of the GQS is that it assigns an objective confidence evaluation on reported genomic regions. Even when there is no genome-wide significant association, GQS values close to 1.0 indicate that the alleles under consideration within the range were measured correctly since they show the expected association between LD and *P*-value. This can support the technical validity of the reported results, which can increase confidence in including an allele in a polygenic risk score analysis or gene set analysis. It can also help justify expanding the sample size to attain statistical significance for the index SNP and the GQS-defined locus and could systematically uncover multiple loci that could benefit from the increased sample size. The more robust analysis by the GQS method can also reduce the variants to be evaluated in replication studies, thus increasing statistical power by predicting SNP association outcomes that are more likely to be true.

When applied to published GWAS meta-analyses summary statistics (Tables 5–8), the GQS supported the robustness of the majority of findings. As an exception, Table 8 shows a surprisingly high number of suspicious regions from the COVID-19 host genomics initiative. These regions have been scrutinized by the COVID-19 HG authors, and they identified a large outlier cohort as the source for these effects. Further support for the reported associations of these suspicious regions was obtained by GQS values for the sample without the outlier cohort, so in summary, we do not question the reported genomic regions. Our GQS results for this study independently identified an underlying issue with the whole-genome meta-analysis, like what we observed in the Asian SCZ GWAS (Table 1).

The GQS method can also be used to reinforce interpretations of GWAS findings. GQS values confirmed the association of schizophrenia with variants in loci containing the cholinergic muscarinic receptor 4 (CHRM4) and dopamine D2 receptor (DRD2) (The Schizophrenia Working Group of the Psychiatric Genomics Consortium *et al.*, 2020). These findings are consistent with the mechanism of antipsychotic drugs that are known (D2) or recently evidenced (CHRM4) to depend on either receptor. The proposal that a muscarinic receptor deficiency characterizes schizophrenia is based in part on *postmortem* gene expression in neurons of schizophrenia patients. The transcriptomes revealed schizophrenia as a diabetes-like condition in the brain (Altar *et al.*, 2005), possibly due to deficient activation of brain receptors for insulin and muscarinic cholinergic receptors (Altar *et al.*, 2008). The specific ability of muscarinic receptor agonists to increase in cultured human neurons the expression of many of the same genes that are decreased in schizophrenia (Altar *et al.*, 2008) is consistent with the antipsychotic efficacy of the muscarinic M1/M4 agonist xanomeline (Shekhar *et al.*, 2008).

SNPs within a locus that contains the muscarinic CHRM4 receptor are highly significant for schizophrenia risk (The Schizophrenia Working Group of the Psychiatric Genomics Consortium *et al.*, 2020), and this locus was technically validated by the GQS of 1.0 (Table 2). A locus containing the DRD2 receptor, the only broadly validated target for treating schizophrenia, is also GWAS-significant, while neither was CHRM1, the other receptor agonist target of xanomeline nor were all other receptor loci except for 10 (Table 3). The GQS values of 1.0 for DRD2 and CHRM4 add validity to the significance levels of the index SNPs and their LD neighborhoods. These results might indicate greater relevance for agonism at the M4 muscarinic receptor versus the M1 receptor subtype to explain the antipsychotic efficacy of xanomeline.

These findings are consistent with recent evidence for the muscarinic receptor as only the second validated target for treating (Shekhar *et al.*, 2008) and possibly contributing to schizophrenia etiology (Altar *et al.*, 2005, 2008).

A comparison between GQS and a recently developed method for QC of GWAS summary statistics, DENTIST (Chen *et al.*, 2021) revealed that in principle the GQS and DENTIST methods work on the same data i.e. the relation between LD and significance, but the target observation is different.

- GQS targets the analysis of whole regions, with an emphasis on the aggregate of genome-wide significant hits, replacing the manual need for visual inspection to support or flag disease risk genes. The GQS was designed, and its algorithm was adjusted to identify the problematic cohorts within a single cohort as well as in meta-analyses.
- DENTIST targets single SNPs in whole-genome summary statistics with the goal of improving fine mapping, conditional analysis, pathway analysis, heritability and genetic correlation. This method is specifically meant for single-cohort association analyses, e.g. not designed for sample size differences between SNPs.
- The GQS method is also available as a user-friendly web tool that does not require an understanding of UNIX, while at present, DENTIST does.

A detailed analysis and results comparing the two methods can be found in the Supplementary Section S2.

In summary, the GQS and DENTIST are useful algorithms that compare the significance of specific associations with the expected significance due to the underlying LD. Discrepancies between the expected and actual outcomes are used to identify suspicious association results.

## 5 Conclusions

The GQS adds confidence to the SNPs in the associated genomic region and removes the need for subjective, visual inspection of associated SNPs. We exclude the incorporation of SNPs that gain signals since these likely reflect one or more additional, independent associations within the same genomic location. The existence of such additional association can be evaluated by a reanalysis of the data by choosing a different index SNP (Supplementary Fig. S8). A second disadvantage is the method’s dependency on the index SNP. For example, if the index SNP is missing power (showing less significance than expected), it will lead to a GQS of 1 even for problematic regions. A third disadvantage is that GQS only works if the region includes SNPs with LD-r2 above 40% (Supplementary Fig. S9). Regions without such SNPs are flagged with a GQS of −1 for manual scrutiny.

A perspective for future uses includes using GQS to find and evaluate GWAS results of borderline significance. Such discoveries may provide a rationale for increasing the sample size to attain sufficient power to test such loci. Finally, the GQS method differentiates reliable, significant GWAS regions according to whether they contain, or do not contain, several technical artifacts. Such artifacts could occur in various steps in GWAS meta-analyses, e.g. errors during QC, imputation or in the meta-analysis setup. This strongly suggests that if applied generally to GWAS results, the GQS method can screen for diverse forms of biases in GWAS and do so without the need for visual inspection. This will be especially relevant for modern multi-site GWAS meta-analyses reporting hundreds of associated regions, where the tedium and subjective nature of visual inspection can be supplanted by objective validation.

## Supplementary Material

btad004_Supplementary_Data

## Data Availability

Most of the data is derived from a source in the public domain or links to download data is incorporated into the article and its online supplementary material.
