# Supplementary material for: GWAS quality score for evaluating associated regions in GWAS analyses
Source: Bioinformatics. 2023 Jan 18;39(1):btad004. doi: 10.1093/bioinformatics/btad004 (PMC9891241; doi:10.1093/bioinformatics/btad004)
Supplement: btad004_Supplementary_Data [file btad004_supplementary_data.docx]

*Table of Contents*

[1. Description simulated data 1](#_Toc113649333)

[2. GQS and DENTIST comparative results. 2](#_Toc113649334)

[a. SCZ GWAS meta-analysis (The Schizophrenia Working Group of the Psychiatric Genomics Consortium et al., 2020) 2](#_Toc113649335)

[b. Neuroticism (Okbay et al., 2016) 2](#_Toc113649336)

[c. Educational attainment (Okbay et al., 2016) 3](#_Toc113649337)

[d. Covid GWAS (COVID-19 Host Genetics Initiative et al., 2021) 3](#_Toc113649338)

[e. Height GWAS (Yenge et al., 2018) 3](#_Toc113649339)

[Reasons for difference: 3](#_Toc113649340)

[Conclusions: 4](#_Toc113649341)

[3. Links to download supplementary figures and tables 4](#_Toc113649342)

[a. Supplementary Tables 4](#_Toc113649343)

[b. Supplementary Figures 1: GQS Hapgen data 4](#_Toc113649344)

[c. Supplementary Figures 2: GQS PGC-SCZ 4](#_Toc113649345)

[d. Supplementary Figures 3: GQS PGC-SCZ asian 5](#_Toc113649346)

[i. **Fig. 3a: GQS PGC-SCZ asian uncorrected** 5](#_Toc113649347)

[**ii.** **Fig. 3b: GQS PGC-SCZ asian corrected** 5](#_Toc113649348)

[e. Supplementary Figures 4: GQS Height GWAS 5](#_Toc113649349)

[f. Supplementary Figures 5: GQS Education attainment GWAS 5](#_Toc113649350)

[g. Supplementary Figures 6: GQS Neuroticism GWAS 5](#_Toc113649351)

[h. Supplementary Figures 7: GQS Covid GWAS 6](#_Toc113649352)

[**i.** **Fig. 7a: GQS Covid GWAS A2** 6](#_Toc113649353)

[**ii.** **Fig. 7b: GQS Covid GWAS B1** 6](#_Toc113649354)

[**iii.** **Fig. 7c: GQS Covid GWAS C2** 6](#_Toc113649355)

[i. Supplementary Figures 8: GQS of regions with multiple signals 6](#_Toc113649356)

[j. Supplementary Figures 9: GQS of regions with -1 6](#_Toc113649357)

[References 7](#_Toc113649358)

Supplementary information

**Title:** GWAS Quality Score for Evaluating Associated Regions in GWAS Analyses

# **Description simulated data^^[[1]](#footnote-1)^^**

From Hapgen (Su et al., 2011) simulated data we randomly selected data from 6,000 European (EUR) and 200 Asian (ASN) individuals and extracted the SNPs of a widely used genotyping platform (Illumina OmniExpress). We then divided the EUR cohort into five subsets (N = 2,000 [cohort 1], 1,000 [cohort 2], 1,000 [cohort 3], 1,000 [cohort 4], 1,000 [cohort 5]) and the ASN cohort into two subsets (N = 100 each). We assigned case and control status randomly to each cohort.

We used cohort 2 to introduce technical errors and association biases. In order to do so, we performed the following steps separately for cases and controls in cohort 2.

- 1. To create autosomal heterozygosity rate deviations in individuals, we changed the heterozygous genotypes to homozygous for all SNPs in 10 selected probands.
  2. To create missingness per individual, a set of 100 probands was selected, and a missing rate of SNPs between 0-10% (from a skewed distribution with higher probability of low missing rates) was introduced.
  3. To create sex errors, we randomly selected 10 (male/female) and swapped their gender assignments.
  4. To create missing SNPs, we randomly selected 2% SNPs of all individuals and introduced missing genotypes by choosing missingness rates between 0-10% (from a skewed distribution with higher probability of low missing rates).
  5. To create Hardy Weinberg disequilibrium per SNPs, we randomly selected 2% of all SNPs and introduced an artificial excess of homozygosity.
  6. To create false positive association SNPs, we selected 20 SNPs and flipped allele1 and allele2 while introducing missingness in these SNPs.

Finally, cases and controls were merged back into a single cohort

# **GQS and DENTIST comparative results.**

The observations from a detailed comparison of GQS and DENTIST underscore the greater validity of GQS when evaluating targeted whole regions, compared to DENTIST.

## **SCZ GWAS meta-analysis (The Schizophrenia Working Group of the Psychiatric Genomics Consortium et al., 2020)**

- - 1. The SCZ GWAS meta-analysis provided 244 genome wide significant regions with strong out-of-sample replication.
    2. The DENTIST method excluded genome-wide significant SNPs within 11 of the 244 regions. In one region (chr5) the entire signal was removed by DENTIST. This would remove two genes (EMB and PARP8) from the list of associated genes and from the list of prioritized genes and eQTL findings (Supplementary Table 20) from the BioRxiv publication as well as from the final publication (Supplementary Table 17 and 20).
    3. In summary, among 2,729 genome-wide significant SNPs in robustly replicating regions (not counting the MHC), 264 would be excluded by DENTIST. The independent index SNPs for these regions did you not show a significant difference in heterogeneity (p>0.97), INFO score (p>0.97), or effective sample size (p>0.99) between flagged and non-flagged SNPs. We cannot identify parameters that indicate suspicion for these SNPs. None have a sign of heterogeneity, or higher missingness, nor a lower info-score or lack of replication. Therefore, we conclude that for at least this meta-analysis, DENTIST appears to be over-conservative for genome-wide significant findings.

## **Neuroticism (Okbay et al., 2016)**

- - 1. There are 10 GWS regions, one of which was flagged with GQS (with a high proportion of exclusions from DENTIST as well), while two other regions were not suitable for GQS. From the remaining 7 regions with strong support from GQS, the DENTIST method excluded 877 SNPs out of all 1,922. This discrepancy illustrates the greater utility of GQS, which evaluates the significance of regions by the aggregate of the SNPs they contain, and their expected association with one another, rather than by the individual association of each SNP as reported by DENTIST.

## **Educational attainment (Okbay et al., 2016)**

- - 1. In educational attainment, there are 73 GWS regions, 71 were suitable for GQS, 66 yielded a high quality GQS value. Four of these were excluded by DENTIST.

## **Covid GWAS (COVID-19 Host Genetics Initiative et al., 2021)**

- - 1. In Covid, 16 regions reported to have genome-wide significance were flagged for inspection by GQS, but DENTIST excluded 5 of these GWAS regions. The flagged regions with GQS of less than 1 have been reported as acceptable after careful inspection by COVID-19 Host Genetics Initiative *et al*., 2021.

## **Height GWAS (Yenge et al., 2018)**

- - 1. In Height GWAS, 1,486 significant and independent regions associated with height, GQS could flag 7 regions that need to be scrutinized. Out of 1,486 regions DENTIST is completely excluding 40 GWS regions and out of these 4 were also flagged by GQS.

## **Reasons for difference:**

- DENTIST mentions in their description of limitations that their method might not work properly if sample sizes differ across SNPs, which might explain our observation that DENTIST can provide an overly conservative algorithmic result. We think different sample sizes are just the reality of modern GWAS meta-analyses and mostly drive regions over-conservatively flagged by DENTIST. We believe that this is important to consider.

## **Conclusions:**

● GQS and DENTIST are useful algorithms that compare the significance of specific associations with the expected significance due to the underlying linkage disequilibrium. Discrepancies between the expected and actual outcomes are used to identify suspicious association results.

● The targets of these two processes are different.

○ GQS evaluates genome-wide significant regions to help identify single genes associated with disease.

○ DENTIST is a quality control for SNPs to improve gene-set analysis. On a separate note, we think that in GWAS meta-analysis, DENTIST may be overly conservative by removing true positive associations.

# **Links to download supplementary figures and tables**

All the supplementary figures and tables (data) can be downloaded from the below link.

<https://personal.broadinstitute.org/sawasthi/share_links/dgnSDwVLjZwNJ2JnRWSPeJUn1hGgvm_Supplementary_Figures1_hapgen.2a.pdf/>

## **Supplementary Tables**

Supplementary Tables 1 to 7 can be downloaded from this link:

<https://personal.broadinstitute.org/sawasthi/share_links/dgnSDwVLjZwNJ2JnRWSPeJUn1hGgvm_Supplementary_Figures1_hapgen.2a.pdf/Supplementary_Tables1-7.xlsx>

## **Supplementary Figures 1: GQS Hapgen data**

Can be downloaded from this link:<https://personal.broadinstitute.org/sawasthi/share_links/dgnSDwVLjZwNJ2JnRWSPeJUn1hGgvm_Supplementary_Figures1_hapgen.2a.pdf/Supplementary_Figures1_hapgen.2a.pdf>

## **Supplementary Figures 2: GQS PGC-SCZ**

Can be downloaded from this link:<https://personal.broadinstitute.org/sawasthi/share_links/dgnSDwVLjZwNJ2JnRWSPeJUn1hGgvm_Supplementary_Figures1_hapgen.2a.pdf/Supplementary_Figures2_pgc_scz_w3.pdf>

## **Supplementary Figures 3: GQS PGC-SCZ asian**

Can be downloaded from this link:

### **Fig. 3a: GQS PGC-SCZ asian uncorrected**

<https://personal.broadinstitute.org/sawasthi/share_links/dgnSDwVLjZwNJ2JnRWSPeJUn1hGgvm_Supplementary_Figures1_hapgen.2a.pdf/Supplementary_Figures3a_pgc_asn_scz_uncorr.pdf>

### **Fig. 3b: GQS PGC-SCZ asian corrected**

<https://personal.broadinstitute.org/sawasthi/share_links/dgnSDwVLjZwNJ2JnRWSPeJUn1hGgvm_Supplementary_Figures1_hapgen.2a.pdf/Supplementary_Figures3b_pgc_asn_scz_corr.pdf>

## **Supplementary Figures 4: GQS Height GWAS**

Can be downloaded from this link:

<https://personal.broadinstitute.org/sawasthi/share_links/dgnSDwVLjZwNJ2JnRWSPeJUn1hGgvm_Supplementary_Figures1_hapgen.2a.pdf/Supplementary_Figures4_height_visscher_2018.pdf>

## **Supplementary Figures 5: GQS Education attainment GWAS**

Can be downloaded from this link:<https://personal.broadinstitute.org/sawasthi/share_links/dgnSDwVLjZwNJ2JnRWSPeJUn1hGgvm_Supplementary_Figures1_hapgen.2a.pdf/Supplementary_Figures5_edu_attain_okbay_2016.pdf>

## **Supplementary Figures 6: GQS Neuroticism GWAS**

Can be downloaded from this link:

<https://personal.broadinstitute.org/sawasthi/share_links/dgnSDwVLjZwNJ2JnRWSPeJUn1hGgvm_Supplementary_Figures1_hapgen.2a.pdf/Supplementary_Figures6_neuroticism_okbay_2016.pdf>

## **Supplementary Figures 7: GQS Covid GWAS**

Can be downloaded from this link

### **Fig. 7a: GQS Covid GWAS A2**

<https://personal.broadinstitute.org/sawasthi/share_links/dgnSDwVLjZwNJ2JnRWSPeJUn1hGgvm_Supplementary_Figures1_hapgen.2a.pdf/Supplementary_Figures7a_covid_A2_rel5.pdf>

### **Fig. 7b: GQS Covid GWAS B1**

<https://personal.broadinstitute.org/sawasthi/share_links/dgnSDwVLjZwNJ2JnRWSPeJUn1hGgvm_Supplementary_Figures1_hapgen.2a.pdf/Supplementary_Figures7b_covid_B1_rel5.pdf>

### **Fig. 7c: GQS Covid GWAS C2**

<https://personal.broadinstitute.org/sawasthi/share_links/dgnSDwVLjZwNJ2JnRWSPeJUn1hGgvm_Supplementary_Figures1_hapgen.2a.pdf/Supplementary_Figures7c_covid_C2_rel5.pdf>

## **Supplementary Figures 8: GQS of regions with multiple signals**

Can be downloaded from this link:<https://personal.broadinstitute.org/sawasthi/share_links/dgnSDwVLjZwNJ2JnRWSPeJUn1hGgvm_Supplementary_Figures1_hapgen.2a.pdf/Supplementary_Figures8_multiple_signals.pdf>

## **Supplementary Figures 9: GQS of regions with -1**

Can be downloaded from this link: <https://personal.broadinstitute.org/sawasthi/share_links/dgnSDwVLjZwNJ2JnRWSPeJUn1hGgvm_Supplementary_Figures1_hapgen.2a.pdf/Supplementary_Figures9_flagged_region.pdf>

# **References**

Chen, W. et al. (2021). Improved analyses of GWAS summary statistics by reducing data heterogeneity and errors. Nature communications,12(1), 7117.

COVID-19 Host Genetics Initiative (2021) et al. Mapping the human genetic architecture of COVID-19. *Nature*, *600*(7889), 472–477.

Lam, M. et al. (2020). RICOPILI: Rapid Imputation for COnsortias PIpeLIne. *Bioinformatics (Oxford, England)*, *36*(3), 930–933.

Okbay, A. et al. (2016). Genetic variants associated with subjective well-being, depressive symptoms, and neuroticism identified through genome-wide analyses. Nature genetics, 48(6), 624–633.

Okbay, A. et al. (2016). Genome-wide association study identifies 74 loci associated with educational attainment. Nature, 533(7604), 539–542.

Su, Z. et al. (2011). HAPGEN2: simulation of multiple disease SNPs. *Bioinformatics* (Oxford, England), *27*(16), 2304–2305.

The Schizophrenia Working Group of the Psychiatric Genomics Consortium et al. (2020) Mapping genomic loci prioritises genes and implicates synaptic biology in schizophrenia. medRxiv

Yengo, L. et al. (2018) Meta-analysis of genome-wide association studies for height and body mass index in ∼700000 individuals of European ancestry. *Hum Mol Genet*, 27(20), 3641-3649.

1. This method is taken from Lam et al., (2020) and slightly modified for this manuscript. [↑](#footnote-ref-1)
